# Artificial grammar learning in vascular and progressive non-fluent aphasias

**DOI:** 10.1016/j.neuropsychologia.2017.08.022

**Published:** 2017-09

**Authors:** Thomas E. Cope, Benjamin Wilson, Holly Robson, Rebecca Drinkall, Lauren Dean, Manon Grube, P. Simon Jones, Karalyn Patterson, Timothy D. Griffiths, James B. Rowe, Christopher I. Petkov

**Affiliations:** a Department of Clinical Neurosciences, University of Cambridge, UK; b Institute of Neuroscience, Newcastle University, UK; c School of Psychology and Clinical Language Sciences, University of Reading, UK; d Medical Research Council Cognition and Brain Sciences Unit, Cambridge, UK

**Keywords:** nfvPPA, Non-fluent variant Primary Progressive Aphasia, PNFA, Progressive Non-Fluent Aphasia, naPPA, nonfluent/agrammatic Primary Progressive Aphasia, PPA-G, Agrammatic Primary Progressive Aphasia, Aphasia, Grammar, Stroke, Frontotemporal dementia, Implicit learning

## Abstract

Patients with non-fluent aphasias display impairments of expressive and receptive grammar. This has been attributed to deficits in processing configurational and hierarchical sequencing relationships. This hypothesis had not been formally tested. It was also controversial whether impairments are specific to language, or reflect domain general deficits in processing structured auditory sequences.

Here we used an artificial grammar learning paradigm to compare the abilities of controls to participants with agrammatic aphasia of two different aetiologies: stroke and frontotemporal dementia.

Ten patients with non-fluent variant primary progressive aphasia (nfvPPA), 12 with non-fluent aphasia due to stroke, and 11 controls implicitly learned a novel mixed-complexity artificial grammar designed to assess processing of increasingly complex sequencing relationships. We compared response profiles for otherwise identical sequences of speech tokens (nonsense words) and tone sweeps.

In all three groups the ability to detect grammatical violations varied with sequence complexity, with performance improving over time and being better for adjacent than non-adjacent relationships. Patients performed less well than controls overall, and this was related more strongly to aphasia severity than to aetiology. All groups improved with practice and performed well at a control task of detecting oddball nonwords. Crucially, group differences did not interact with sequence complexity, demonstrating that aphasic patients were not disproportionately impaired on complex structures. Hierarchical cluster analysis revealed that response patterns were very similar across all three groups, but very different between the nonsense word and tone tasks, despite identical artificial grammar structures.

Overall, we demonstrate that agrammatic aphasics of two different aetiologies are not disproportionately impaired on complex sequencing relationships, and that the learning of phonological and non-linguistic sequences occurs independently. The similarity of profiles of discriminatory abilities and rule learning across groups suggests that insights from previous studies of implicit sequence learning in vascular aphasia are likely to prove applicable in nfvPPA.

## Highlights

• Patients with Broca's aphasia and nfvPPA show implicit artificial grammar learning.
• Both groups displayed global deficits compared to controls but improved with practice.
• Neither group was disproportionately impaired on more complex ordering relationships.
• Learning did not transfer between otherwise identical speech and tone-based grammars.
• Separate linguistic and non-linguistic sequence processing was maintained in aphasia.

## Graphical abstract

**Figure:**
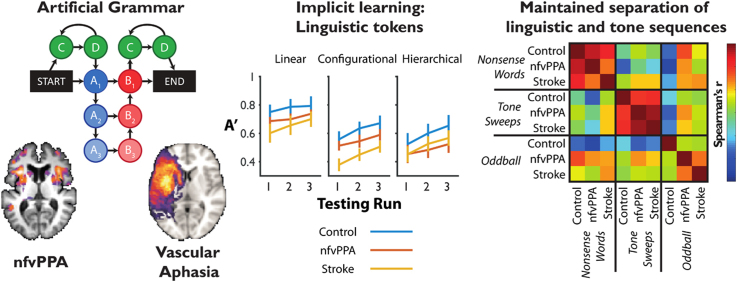

## 1 Introduction

Aphasia is an impairment of speech and language that often leaves other cognitive and intellectual capacities preserved. Patients with non-fluent aphasias due to frontal lobe damage exhibit significant impairments in grammar (Caramazza and Zurif, 1976, Caplan et al., 1985, Berndt et al., 1996). The grammatical impairments in comprehension and production are separable, but tend to be highly correlated (Berndt et al., 1983), suggesting that they stem from disruption of core syntactic processes rather than processes such as memory, executive function or motor function (Wilson et al., 2011). The deficits are phenomenologically similar in patients with damage due to neurodegeneration (non-fluent variant Primary Progressive Aphasia, nfvPPA) and stroke (‘Broca's aphasia’), however detailed analysis of speech output has revealed somewhat differential impairments (Patterson et al., 2006, Thompson et al., 2013). Impairments of receptive abilities have not been compared in similar detail.

Beyond these linguistic deficits, patients with aphasia also display auditory domain general processing deficits that are not specifically related to language (Caramazza and Zurif, 1976, Christiansen et al., 2010a, Dominey et al., 2003, Geranmayeh et al., 2014b, Goll et al., 2010, Grube et al., 2016, Grube et al., 2012, Patel et al., 2008, Zimmerer et al., 2014a, Zimmerer et al., 2014b, Zimmerer and Varley, 2015). Such studies have raised the possibility that deficits in structured sound processing may play a prominent role in language disorders, but the nature and extent of these deficits remains unclear. It also remains unclear whether impairments in aphasia are specific to the speech domain (Conway and Pisoni, 2008), or also apply to non-linguistic auditory sequences (Christiansen et al., 2010). One study identified impairments in implicit musical sequence learning in vascular aphasia (Patel et al., 2008), but direct comparisons outside of a musical framework are lacking. If artificial grammar learning tasks tap into domain general (rather than language specific) processes, one might expect rule acquisition to generalise from sequences of nonsense words to identically structured sequences of other sounds, such as tones.

It has been commonly held that grammatical impairments are specific to complex linguistic constructs such as hierarchical relationships and the passive voice (Goodman and Bates, 1997, Grodzinsky, 2000), but there is limited evidence for such dissociations (Zimmerer et al., 2014a, Zimmerer et al., 2014b). By contrast, some studies suggest that the processing of adjacent relationships may be disproportionately impaired by frontal lesions involving motor association cortex (Opitz and Kotz, 2012). Recent studies examining artificial grammar learning in agrammatic aphasia secondary to stroke have focussed on linear sentential structures with varying transitional probabilities (Schuchard and Thompson, 2017). A key outstanding question, therefore, is whether agrammatic aphasia is characterised specifically by deficits for more complex linguistic structures or rather by a more global impairment in processing structured auditory sequences (Berndt, 2000).

Artificial grammar learning tasks are particularly well suited for delineating competence in structured sequence processing, as they focus on ordering relationships in the absence of other cues (e.g., semantics, phonology or pragmatics). They test learning of the rules governing the order in which stimuli occur in a sequence (Reber, 1967). Participants are typically exposed to sequences of stimuli that follow certain rules, so that the ordering relationships between the sequence elements can be learned implicitly. They are then tested with novel sequences that are either consistent with these rules or that violate them in some way, to assess learning. The implicit nature of these tasks allows the testing of a wide range of participants, including patients with aphasia. Unlike natural language tasks, it is possible to present structurally identical sequences comprised of different tokens, for example nonsense words or non-linguistic tone stimuli, to assess the contribution of phonological processing. Finally, artificial grammars with multiple levels of complexity can be used to quantify how well participants are able to learn increasingly complex rules, which may more closely reflect those in natural language grammars (Romberg and Saffran, 2013, Wilson et al., 2015).

The ability to process auditory sequences, even when stimuli are meaningless, is strongly linked with linguistic proficiency (Gómez and Gerken, 2000, Conway and Pisoni, 2008, Conway et al., 2010, Frost et al., 2015). Neuroimaging studies have demonstrated that artificial grammar processing engages a left-lateralised network of frontal, temporal and parietal brain areas similar to the set of regions involved in syntactic operations during natural language tasks (Friederici et al., 2000, Ni et al., 2000, Friederici and Kotz, 2003, Petersson et al., 2004, Forkstam et al., 2006, Friederici et al., 2006, Hickok and Poeppel, 2007, Bahlmann et al., 2008, Makuuchi et al., 2009, Folia et al., 2011, Friederici, 2011, Fedorenko et al., 2012, Petersson et al., 2012a, Petersson et al., 2012b, Petersson et al., 2012a, Petersson et al., 2012b) and is associated with developmental language impairment (Evans et al., 2009).

The sequence processing ability of patients with non-fluent aphasia has not been systematically compared across aetiologies. Non-fluent variant Primary Progressive Aphasia (nfvPPA), also variously known as Progressive Non-Fluent Aphasia (PNFA), nonfluent/agrammatic Primary Progressive Aphasia (naPPA), and Agrammatic Primary Progressive Aphasia (PPA-G), is an adult onset neurodegenerative aphasia characterised by agrammatism and speech apraxia (Gorno-Tempini et al., 2011). It is in many ways the neurodegenerative equivalent of Broca's aphasia, though some differences do exist in the pattern of speech output impairment (Patterson et al., 2006). The majority of cases are associated with primary tau pathology but a significant minority have TDP-43 related disease (Kertesz et al., 2005, Josephs et al., 2006, Knibb et al., 2006a, Knibb et al., 2006b, Mesulam et al., 2014). nfvPPA typically leads to subtle structural neuroimaging changes in left inferior frontal and insular cortex (Gorno-Tempini et al., 2004), which correlate with clinical severity (Rogalski et al., 2011). Chronic non-fluent aphasia due to stroke (Broca's aphasia) results in a similar clinical phenotype of agrammatism and apraxia of speech. The left frontal tissue damage is stable, with partial clinical improvement over time (Kertesz and McCabe, 1977). The extent and pace of this improvement is variable and depends strongly on the integrity of the underlying white matter (Price et al., 2010, Seghier et al., 2016). Better understanding of the abilities of participants with similar symptoms arising from very different aetiologies could provide valuable insights into the neurobiological underpinnings of domain-general and language-related processes, and inform treatment strategies (Brownsett et al., 2014, Geranmayeh et al., 2014a, Geranmayeh et al., 2014b).

In the present study, patients with nfvPPA, non-fluent aphasia due to stroke, and matched controls were tested on their implicit learning of a mixed-complexity artificial grammar, combining sequencing relationships of increasing complexity using nonsense words or tones. We aimed to test the following linked hypotheses:

1) Rule acquisition differs when structurally identical sequences are comprised of nonsense words rather than non-linguistic tones.
2) Artificial grammar learning ability is similar in patients with vascular and neurodegenerative aphasia.
3) Grammatical impairments in aphasic patients are disproportionately greater for complex, configurational or hierarchical, sequencing operations.
4) Patients with aphasia can improve their ability to detect grammatical disruptions with repeated implicit training.

## 2 Methods

### 2.1 Participants

**Fig. 1:**
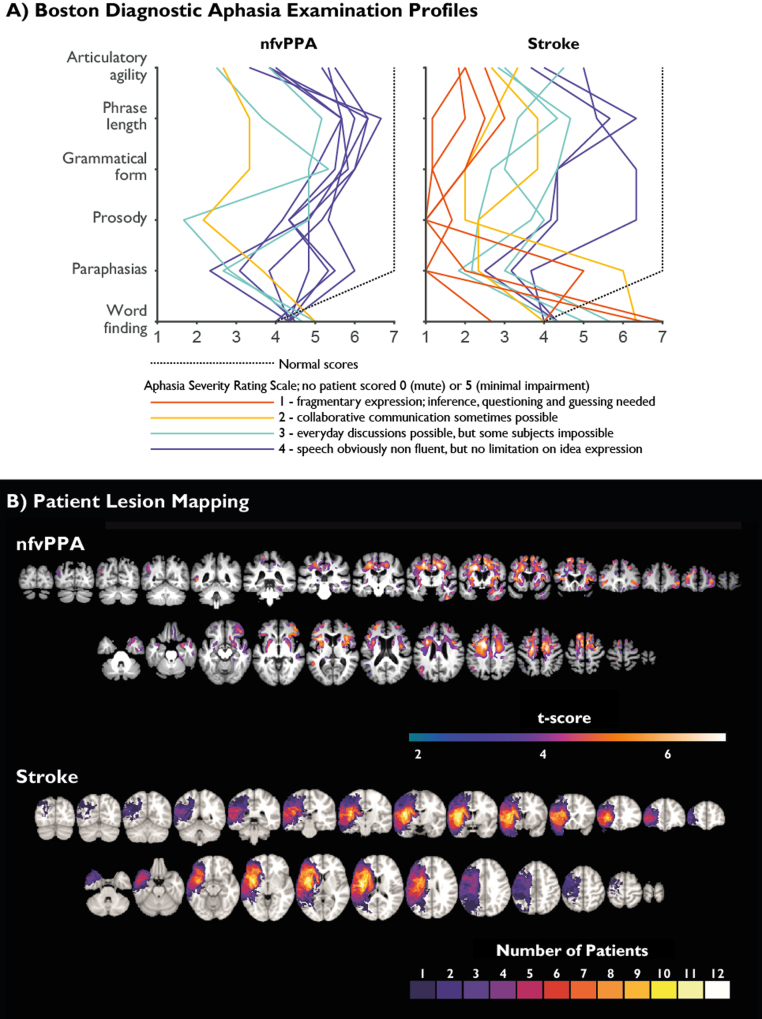
A) Boston Diagnostic Aphasia Examination Profiles for nfvPPA and stroke groups. Normal values illustrated as broken black line. Colour coding of individual profiles based on Aphasia Severity Rating Scale; 1 = red, 2 = magenta, 3 = yellow, 4= blue. No patients had an ASRS of 0 (no usable speech or auditory comprehension) or 5 (minimal discernible handicap). B) Upper: Voxel based morphometry of nfvPPA vs age-matched healthy controls. Coloured regions demonstrate cluster-wise significance at FWE<0.05 with a cluster defining threshold of 0.001 for either grey or white matter volume. Lower: lesion overlap map for the stroke group. (For interpretation of the references to color in this figure legend, the reader is referred to the web version of this article.)

Three groups of participants were recruited. Demographics of the groups are outlined in Table 1. All patients were right handed. One control was left handed. Thirteen patients with mild to moderate nfvPPA were identified from specialist cognitive clinics led by authors JBR and TDG according to consensus diagnostic criteria (Gorno-Tempini et al., 2011). These criteria were strictly applied; particular care was taken to exclude non-fluent patients who had lexical difficulties, in order to select patients most likely to have underlying Tau or TDP-43 related pathology preferentially involving left frontal lobes, rather than Alzheimer-type pathology of parietal lobes (Rogalski et al., 2011, Rohrer et al., 2012, Sajjadi et al., 2012, Sajjadi et al., 2014, Mandelli et al., 2016). Three patients were excluded on the basis of yes/no response confusion (a common early symptom in nfvPPA that might otherwise have reduced our power to detect language specific effects), resulting in 10 complete nfvPPA datasets. On the short form of the Boston Diagnostic Aphasia Examination (BDAE) (Goodglass et al., 1983) all patients scored 10/10 for responsive naming, 12/12 for special categories, at least 15/16 for basic word discrimination and at least 9/10 for following complex commands. While nfvPPA exists on a spectrum, with differing ratios of speech apraxia and agrammatism, all of our patients displayed some degree of impairment of expressive grammar in free speech, and all but two displayed impairment of receptive grammar as measured by the sentence comprehension task on the ‘verb and sentences test’ (VAST) (Bastiaanse et al., 2003) (mean 87.5%, range 70–100%). Similarly, the patients varied in their degree of expressive agrammatism, but none was completely unimpaired. Samples of speech from the participants are available in supplementary materials, and speech profiles are shown in Fig. 1A. BDAE profiles were independently rated by authors TEC, HR and KP. Inter-rater reliability for grammatical form was high, with pairwise Pearson correlations of 0.87, 0.85 and 0.85. Areas of significant grey or white matter loss are shown in Fig. 1B, upper panel.

**Table 1:** Subject demographics. Mean (s.d., range). Age leaving education is reported as it is a better measure of highest scholastic attainment than number of years in study. No individuals were mature students.

	Control	nfvPPA	Stroke
Number	11	10	12
Age	69 (8, 54–79)	73 (7, 63–82)	60 (11, 33–74)
Age leaving education	18 (2, 15–22)	18 (3, 15–25)	20 (4, 15–26)
Years of musical training	2 (3, 0–10)	1 (1, 0–3)	3 (5, 0–13)

**Fig. S1:**
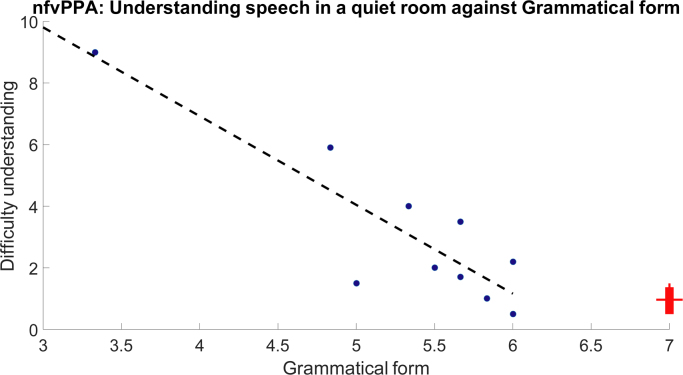
A plot for nfvPPA of difficulty ‘Understanding speech in a quiet room’ against ‘Grammatical form’ from the BDAE. Individual patients are shown as blue circles. A boxplot of control ratings is shown in red. The dashed trend line had an r^2^ of 0.778 (p<0.001).

It is widely recognised that patients with nfvPPA report difficulties with understanding speech (Goll et al., 2010, Cope et al., 2014, Grube et al., 2016). We asked the patients in this study to complete visual analogue scales assessing their difficulty with ‘Understanding speech in a quiet room’, ‘Telling the direction a sound is coming from’, ‘Understanding speech in a noisy restaurant’, ‘Hearing announcements at a bus or rail station’ as well as ‘How loud do people tell you your TV is?’ Compared to a matched group of controls, patients differed only in reporting more difficulty with ‘Understanding speech in a quiet room’ (p=0.02). Of the Boston Diagnostic Aphasia Examination (BDAE) sub-scores, this difficulty was strongly correlated only with ‘Grammatical form’ (r^2^=0.778, p<0.001, Supplementary Figure 1).

Twelve patients with non-fluent aphasia due to left sided stroke were recruited from a volunteer database administered by author HR, supplemented by the identification of incident cases by regional research networks. Recruitment criteria were: a single stroke of at least six months chronicity resulting in at least one month of non-fluent aphasia, with MRI evidence of involvement of either left inferior trigone or operculum. Samples of speech from the participants are available in supplementary materials, and speech profiles (triple marked by authors TEC, HR and KP) are shown in Fig. 1A. On the whole, the stroke group had more severe language impairments than the nfvPPA group. All had some degree of impairment of grammatical form in free speech, and all but two had impairment of receptive grammar on the VAST (mean 70%, range 40–100%). Lesion overlap maps are shown in Fig. 1B, lower panel.

Care was taken to recruit an appropriate control group. During development of the artificial grammar, extensive piloting developed structures for which learning was least influenced by years of education or performance on global cognitive tests. Nonetheless, it is important to minimise this potential confound by avoiding the use of biased volunteer panels, which tend to preferentially recruit highly educated individuals with supra-normal motivation in research tasks. Therefore, we recruited 8 neurological controls with either chronic inflammatory demyelinating polyneuropathy or multifocal motor neuropathy with conduction block, and three spouses of patients with nfvPPA, resulting in 11 control datasets. These individuals were chosen to represent a cohort of age-matched individuals with healthy brains and similar levels of habitual neurological contact to the patient groups. All scored normally on the Addenbrookes Cognitive Examination – Revised (ACE-R) (mean 96/100, range 92–99) and Raven's progressive matrices (mean 47/60, range 37–60).

**Fig. S2:**
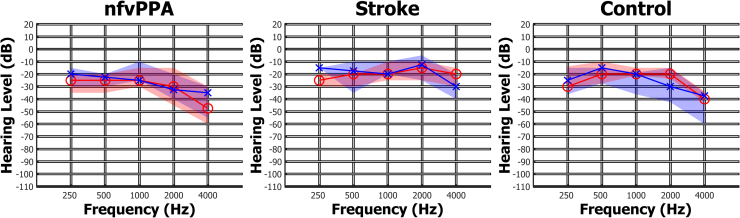
Composite pure tone audiograms for each group. Red circles joined by lines denote the median auditory threshold in the right ear; blue crosses joined by lines the median auditory threshold in the left ear. Shaded areas represent the inter-quartile range of auditory acuity in the right (red) and left (blue) ears.

It was possible to perform pure tone audiometry in all patients with nfvPPA, 9 of the 12 patients with stroke aphasia and 8 of the 11 neurological controls. This demonstrated that the groups had well matched and age-appropriate auditory acuity (Supplementary Figure 2).

### 2.2 Stimuli

**Fig. 2:**
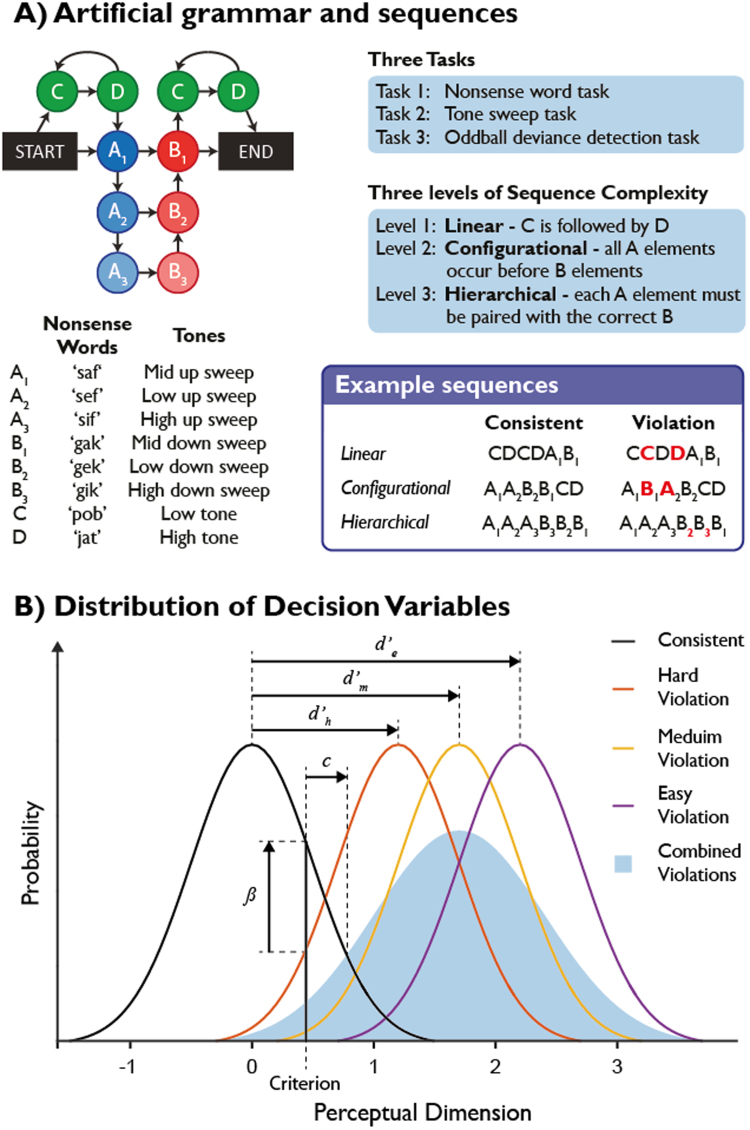
A: Artificial grammar structure and stimuli. B: Cartoon illustrating the distribution of parametric decision variables for an hypothetical experiment with easy, medium and hard to detect rule violations. d′ represents the single subject discriminability of each rule violation, while c and β represent different measures of bias (i.e. in our case the tendency to say that a sequence is grammatical if there is no evidence to the contrary). Each rule violation has its own d′ measure, reflecting its respective discrimination difficulty, while the bias measures apply to the experimental context as a whole.

The Artificial Grammar (AG) used here generates sequences of stimuli from 8 unique elements (Fig. 2A). These sequences are governed by a number of rules of increasing complexity. Rule 1) if a ‘C’ element occurs it must be immediately followed by a ‘D’ element. This represents a simple, invariant linear relationship between two adjacent sequence elements, and will henceforth be referred to as the ‘linear’ rule. Rule 2) all of the ‘A’ elements in the sequence must occur before all of the ‘B’ elements. This is a more complex rule, requiring the participants to recognise a general property of the sequences, and will henceforth be referred to as the ‘configurational’ rule (Zimmerer and Varley, 2015). Rule 3) each ‘A’ element type must be paired with the appropriate ‘B’ elements in embedded relationships (e.g., A_1_[A_2_[A_3_B_3_]B_2_]B_1_). This complex operation requires tracking both the number and the order of the ‘A’ elements and matching these to the subsequent ‘B’ elements, and is referred to as the ‘hierarchical’ rule.

Sequences consistent with the AG are generated by following any path of arrows from start to end in the illustrated state transition graph (Fig. 2A). Ten consistent sequences were used for the exposure phase of the experiment (Table 2). These sequences were of variable length and contained all of the legal transitions possible with the AG. The remaining subset of consistent sequences generated by the AG were kept for the subsequent testing phase, to allow us to present novel, previously unheard sequences (Table 2). During the testing phase, the participants were presented with 4 repetitions each of 6 consistent and 6 violation sequences, in a pseudo-random order. All test sequences were six elements long, meaning that sequence duration could not be used as a cue by participants. The six violation sequences contained two sequences with violations of each of the three AG rules (Table 2). This design allowed us to identify the specific features of the sequences to which the participants were sensitive.

**Table 2:** Exposure and testing sequences.

Exposure Sequences	Testing Sequences		
	A_1_A_2_A_3_B_3_B_2_B_1_	Consistent	Familiar
A_1_A_2_A_3_B_3_B_2_B_1_	A_1_B_1_CDCD	Consistent	Familiar
CDA_1_B_1_CD	CDA_1_B_1_CD	Consistent	Familiar
A_1_B_1_CDCD	CDA_1_A_2_B_2_B_1_	Consistent	Novel
A_1_A_2_B_2_B_1_	A_1_A_2_B_2_B_1_CD	Consistent	Novel
CDA_1_B_1_	CDCDA_1_B_1_	Consistent	Novel
A_1_B_1_CD	C**C**D**D**A_1_B_1_	Violation	Violates Rule 1
CDCDA_1_B_1_CD	**DC**A_1_B_1_**DC**	Violation	Violates Rule 1
CDA_1_A_2_A_3_B_3_B_2_B_1_	CD**B**_1_**A**_1_CD	Violation	Violates Rule 2
A_1_A_2_A_3_B_3_B_2_B_1_CD	A_1_**B**_1_**A**_2_B_2_CD	Violation	Violates Rule 2
A_1_A_2_A_3_B_3_B_2_B_1_	A_1_A_2_B_**1**_B_**2**_CD	Violation	Violates Rule 3
	A_1_A_2_A_3_B_**2**_B_**3**_B_1_	Violation	Violates Rule 3

We tested participants with identically structured sequences of both naturally spoken consonant-vowel-consonant (CVC) nonsense words and non-linguistic tone stimuli. The stimuli were designed to provide acoustic cues to highlight the relationships between some of the key sequencing relationships, as follows. In both the CVC and tone experiments, the ‘A’ and ‘B’ elements fell into distinct acoustic categories. In the CVC experiment the ‘A’ elements all took the form “s-vowel-f” (e.g., “sif”) while the ‘B’ elements were “g-vowel-k” (e.g., “gik”). In the tone experiment the ‘A’ elements were all upwards pitch sweeps while the ‘B’ elements were downward sweeps. Furthermore, the A_X_-B_X_ relationships that are critical to Rule 3 were highlighted by the presence of the same vowel sounds in the nonsense word experiment (i.e., A_1_ and B_1_ both contain the central vowel ‘a’) or the tone height in the tone experiment (i.e., both the A_1_ and B_1_ pitch sweeps are centred on the same frequency). To ensure that the participants learned aspects of the AG during the exposure phase, rather than simply responding to acoustical properties of the stimuli, we designed the tone stimuli to avoid linear increases or decreases in pitch in the A_1_A_2_A_3_ or B_3_B_2_B_1_ parts of the sequences. Instead, the centre frequencies of the tone sweeps in such a sequence would be ‘mid-low-high-high-low-mid’. The ‘C’ and ‘D’ elements in the nonsense word experiment were designed to be clearly phonetically distinct from the ‘A’ and ‘B’ stimuli, and in the tone experiments they were continuous pure tones of high or low pitch. Example sequences are available to listen to in the supplementary materials.

The nonsense words were produced by a female speaker, recorded with an Edirol R-09HR (Roland Corp.) sound recorder, and combined into exposure and testing sequences using Matlab (100 ms inter-stimulus intervals, ISI). The average duration of the nonsense words was 477 ms (standard deviation = 7 ms). The tone stimuli were generated using Matlab. The low tone sweeps were linear sweeps between 100 and 150 Hz (i.e., A_2_ began at 100 Hz and increased to 150 Hz, B2 began at 150 Hz and decreased to 100 Hz). The middle tone sweeps spanned 200 to 300 Hz and the high tone sweeps spanned 400 to 600 Hz. The C and D stimuli were pure tones at 350 and 800 Hz respectively. The duration of all tones was 450 ms, and these were combined into sequences with ISIs of 100 ms. Stimuli were presented through Sennheiser HD250 linear 2 headphones, driven by either an Edirol UA-4X or Behringer UCA 202 external sound card. The amplitudes of all stimuli were root-mean-square (RMS) balanced, and sequences were initially presented to participants at ~75 dB SPL (calibrated with an XL2 sound level meter, NTI Audio). At the start of the exposure phase, participants were asked if this volume was comfortable and clearly audible and, if not, were allowed to freely adjust the volume to their preference.

Exposure sequences and test sequences for the CVC and tone languages were presented in exactly the same fixed pseudo-random order. There were no differences between the orders of sequences in the CVC and tone runs; only the sound tokens used to represent each element in the artificial grammar differed.

### 2.3 Procedure

The experimental procedure and instructions given to participants were tightly constrained, to ensure that explicit learning strategies and the effect of receptive language difficulties were minimised. The exact wording of the instruction is included in supplementary materials.

Participants were exposed to the CVC language for five minutes. During this time they were simply instructed to listen to the language and to pay attention to the order of the words (the exact script for the instructions is available as supplementary material). They were then tested by being asked to decide whether 48 individual sequences (Table 2) were correct (i.e. consistent with the artificial grammar) or incorrect (i.e. violated the artificial grammar in some way). Participants were able to express their decision either by pressing a button on a keyboard or custom made response box, or by pointing to yes or no on a piece of paper; whichever they found easiest. At the end of a run, general overall feedback was provided with smiley to sad faces (Wong and Baker, 1988) according to overall percentage correct, along with the performance descriptor ‘Great!’ (>60%), ‘Well’ (55–60%), ‘OK’ (45–55%), or ‘Badly’ (<45%). This exposure-test cycle was then repeated in an identical fashion for the tone language. Again, they were explicitly instructed that the important thing was the order of the sounds.

After a short break, participants were then re-exposed to the CVC language for three minutes, before being re-tested. Sequences for both exposure and testing were presented in a different fixed pseudo-random order on each repetition. At the end of this run, feedback was provided relative to the previous CVC run with faces paired with the descriptors ‘Much Better’ (>110% of previous score), ‘Better’ (105–110%), ‘Same’ (90–105%), or ‘Worse’ (<90%). This procedure was then repeated for the tone language.

After a longer break, during which tea and biscuits were provided, participants completed a personal details questionnaire, which included questions about musical training and handedness (all patients were right handed). Patients then undertook the short form of the Boston Diagnostic Aphasia Examination (Kaplan, 1983) and the first half of the sentence comprehension section of the Verbs and Sentences Test to assess receptive grammar (Bastiaanse et al., 2003); controls completed an ACE-R and were tested on matrix reasoning (Raven, 1960), similarly demanding tasks of similar duration. Participants then completed another three minute exposure and test session on the CVC and tone languages, for a total of three testing sessions for each language.

Finally, each participant was re-exposed to the CVC language for three minutes, but the testing session that followed was replaced with an ‘oddball’ task. Participants were told that in this final test the ‘incorrect’ sequences were wrong in a different way, but were not explicitly instructed that they were listening for novel tokens. Where an ordering violation would first have occurred in an ‘incorrect’ sequence, the CVC token was replaced by a novel, previously unheard, oddball element (‘fen’, ‘muz’, ‘rol’, ‘dut’, ‘boz’ or ‘cav’). In this way, we were able to assess whether differential performance on the three grammatical rule types was related to other undesired effects such as stimulus ordering.

All individuals undertook all study procedures on a single day, to ensure that differential patterns of performance consolidation during sleep did not confound our findings. The study procedures took up to four hours, including breaks.

### 2.4 Stroke lesion mapping

The lesioned area of each brain was manually defined on every slice of each patient's 3T T1-weighted MRI scan in FSL, resulting in a 3D lesion mask. The resulting image was then registered to the standard MNI152 brain using FLIRT (FMRIB's Linear Image Registration Tool (Jenkinson and Smith, 2001); affine transformation model, 12 degrees of freedom). This registration matrix was used to register the patient's lesion mask to the standard space, from which a standardised lesion volume was computed in Matlab. Regions of interest in the left hemisphere (frontal inferior trigone, frontal inferior operculum, rolandic operculum, putamen and caudate) were identified using the aal atlas in SPM12, and the percentage of each sub-region that was lesioned was extracted for analysis (Supplementary Table 1).

### 2.5 nfvPPA atrophy mapping

Nine of the patients in the nfvPPA group underwent a 3T volumetric T1 MRI scan. From a database of healthy control scans from the same scanner, an age-matched normative sample of 36 individuals was selected by finding the four nearest-neighbours to each patient in terms of age, excluding duplication (mean age of these controls was 73 years). After segmentation, the nine nfvPPA scans and nine nearest-neighbour controls were used to create a DARTEL template. This was then applied to the remaining 27 controls. Resultant images were normalised to MNI space in SPM12 with an 8 mm smoothing kernel, and separate statistical comparisons were performed for grey and white matter, with total intracranial volume and age as covariates (Fig. 1B).

As for the stroke lesion mapping, regions of interest in the nfvPPA patients were then identified using the aal atlas, normalised to MNI space. As distinct from the lesion analysis in the stroke cases, these regions were defined bilaterally. The grey matter volume in each region was then extracted by applying these regions to each individual's modulated, warped grey matter segmentation and correcting for total intracranial volume.

### 2.6 Analysis

Performance metrics for analysis were based on signal detection theory (Stanislaw and Todorov, 1999). Standard signal detection measures of discriminability and bias rely on the underlying trial difficulty within a run being constant. In our artificial grammar, it was expected that violations of the linear relationship (complexity level 1) would be easier to detect than configurational violations (complexity level 2), which in turn might be easier to detect than violations of the hierarchical structure (complexity level 3) (see Fig. 2B). To accommodate this, we separately calculated d′ and the non-parametric equivalent A′, based on a comparison of performance on each of the three types of violation sequence to all of the consistent sequences. A single value for bias measures was also calculated based on the combined distribution of violation sequences (Fig. 2B). Hit and false alarm rates of 1 were replaced with (n-0.5)/n (where n is the number of trials), and those of 0 with 0.5/n (Macmillan and Kaplan, 1985, Stanislaw and Todorov, 1999). The non-parametric analogues of these signal detection metrics, A′ for discriminability and β’’ for bias, were used for the primary analysis.

All statistical analyses were performed in Matlab R2015b with the Statistics and Machine Learning Toolbox unless otherwise specified. Differences from chance performance in both discriminability measures (A′ and d′) and measures of bias (ln(β), c and β’’) were assessed for each group and condition separately using one-sample Wilcoxon signed rank tests (the non-parametric equivalent of the one sample *t*-test).

The effects of group and rule complexity on discriminability were assessed with three separate repeated measures ANOVA tests (one for each test type: CVC, tones and oddball), with the factor ‘participant number’ nested within ‘group’. This parametric statistical test was employed because there is no appropriate non-parametric test for repeated measures designs of the kind employed here; the Friedman test does not allow multiple groups to be compared. Significant results were explored with post-hoc comparisons of population marginal means.

The degree of learning across exposure-test pairs was assessed by fitting a general linear model in Minitab 17 for the CVC and tone languages. The response variable was discriminability (A′) and the factors were ‘participant number’ (nested within ‘group’), ‘rule type’, and ‘run number’. Significant results were explored with post-hoc Tukey's range tests.

To assess whether the same rules were learned by participants between task types (CVC vs tones vs oddball), and by extension whether learning of the same artificial grammar was transferrable across token types (CVC vs tones), Spearman correlation matrices were constructed based on performance patterns by sequence for each group. From these, hierarchical cluster analysis was performed to construct dendrograms representing the similarity of performance pattern across test and group, using a ‘farthest neighbour’ linkage method with a data-driven inconsistency coefficient (The Mathworks Inc, 2015).

Exploratory regression analyses were performed in Minitab 17. As there were a large number of variables measured for each subject, stepwise regression was undertaken to determine those variables that best predicted artificial grammar learning. This is an automated process to identify a useful subset of predictors by sequentially adding and removing predictors until an optimal model is obtained. Software default alpha-to-enter and alpha-to-remove values of 0.15 were used, with confirmatory analyses at 0.1/0.15 and 0.1/0.1 yielding identical results. Three separate sets of stepwise regressions were performed; one to explore possible correlations between rule discriminability and neuropsychological and language measures, a second to explore correlations between rule discriminability and stroke lesion site, and a third to explore correlations between rule discriminability and grey matter volume in nfvPPA. The potential continuous predictors included in the first model set were Age, Raven's Progressive Matrix score, years of musical training (which we hypothesised might impact tone language difficulty), sentence comprehension (from the Verbs and Sentences Test), and overall aphasia severity (Aphasia Severity Rating Score). For the second set, the potential continuous predictors were age, the proportion of each region of interest lesioned, total lesion volume, and the number of years since stroke. For the third, potential predictors were age, grey matter volume summed across regions of interest in each hemisphere, and corrected whole brain grey matter volume.

## 3 Results

**Fig. 3:**
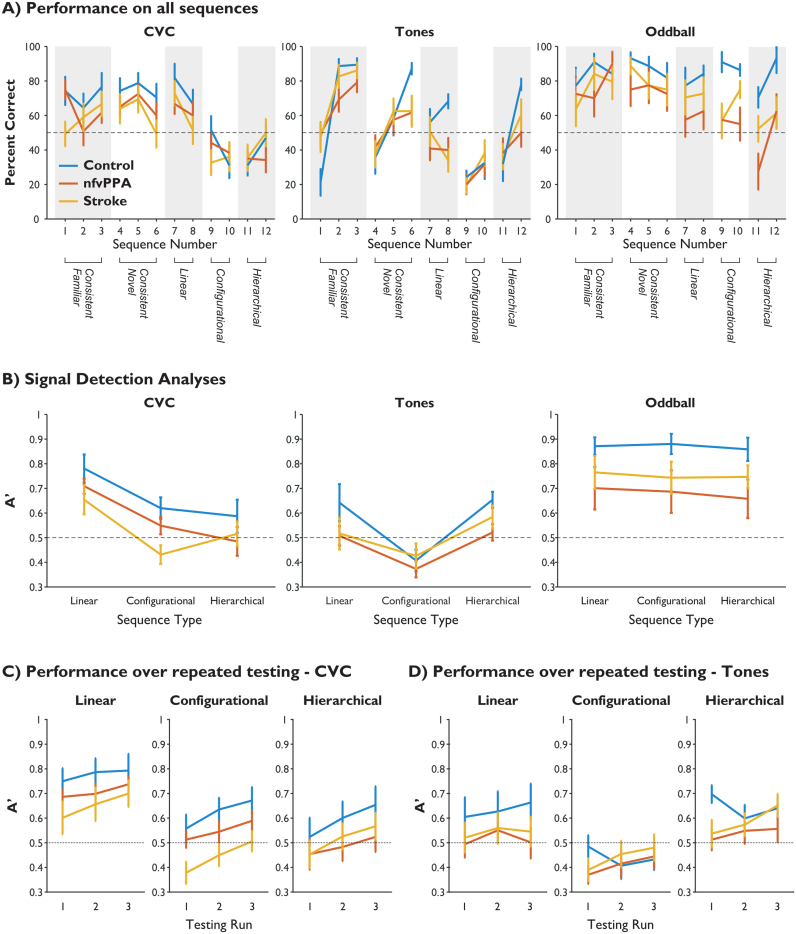
Group performance on sequence identification. Dashed lines represent chance performance. Error bars represent group-wise standard error of the mean. Controls are in blue, nfvPPA in red and stroke in orange. A) Proportion of correct responses for each testing sequence by group and task. Sequences 1–3 were consistent with the grammar and familiar from the exposure phase, while 4–6 were consistent and novel. Sequences 7–12 contained violations of the types indicated (see Table 2). B) Discriminability of each rule type by group for each language type. C) Overall discriminability by group and run number for the CVC language (improving performance by run represents learning over time). D) Overall discriminability by group and run number for the tone language. (For interpretation of the references to color in this figure legend, the reader is referred to the web version of this article.)

**Fig. 4:**
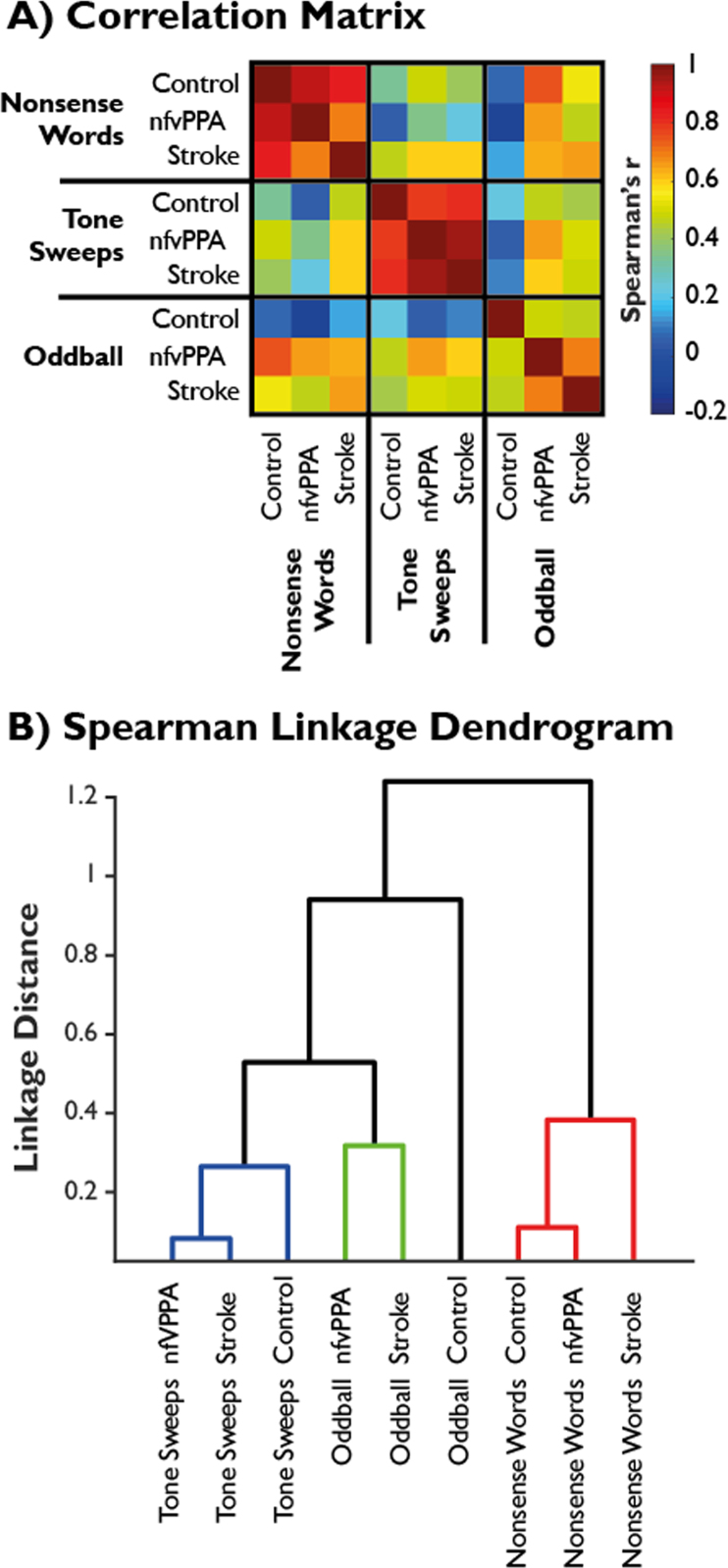
A) Spearman rank-order correlation matrices. B) Linkage based on hierarchical cluster analysis of Spearman correlations. Three clusters emerge with a linkage distance cutoff of 0.5, and are indicated in colour groupings (blue, green and red). (For interpretation of the references to color in this figure legend, the reader is referred to the web version of this article.)

Raw performance for each individual sequence is shown in Fig. 3A, and the results of the signal detection theory analysis are illustrated in Fig. 3B. The hierarchical cluster analysis is illustrated in Fig. 4. Performance did not differ between sequences heard during exposure and test phases (Fig. 3A sequences 1–3) and those that were novel during the test phase (Fig. 3A sequences 4–6), so these were collapsed. Results are presented for the non-parametric discrimination measure A′, but the same pattern of findings was present for the parametric equivalent d′ (Supplementary Tables 2 and 3).

For the CVC language, discriminability as measured by A′ showed significant main effects of rule complexity and group, but no group x rule interaction or consistent inter-individual differences (Fig. 3B, Table 3A). Post-hoc comparison of marginal means indicated that the group difference is driven by the control participants performing significantly better than participants in the stroke group (p=0.0058). Participants with nfvPPA performed at an intermediate level, and were not statistically different from either controls (p=0.14) or stroke (p=0.50). Further, all groups performed better at detecting violations of linear grammatical rules than configurational (p=0.0001) or hierarchical (p=0.0001), which in turn did not differ (p=0.99).

**Table 3:** A and B: Repeated measures ANOVAs of group against rule for the non-parametric discriminability measure A′ (panel A; corresponding to Fig. 3A) and bias (panel B; corresponding to Fig. 3B), with participant number as a nested factor within group. C: The general linear model assessing learning across runs for the non-parametric discriminability measure A′ (corresponding to Fig. 3C (CVC) and 3D (Tones)).

A - Fig. 3A	Rule complexity	Group	Group x complexity	Participant	
CVC	**F(2,60) = 14.8**	**F(2,60) = 4.11**	F(4,60) = 0.70	F(30,60) = 1.42	
	**p<0.0001**	**p=0.0264**	p=0.5933	p=0.1246	
Tones	**F(2,60) = 9.05**	**F(2,60) = 7.13**	F(4,60) = 0.49	F(30,60) = 0.32	
	**p=0.0004**	**p=0.0029**	p=0.7421	p=0.9995	
Oddball	F(2,60) = 0.51	F(2,60) = 2.59	F(4,60) = 0.16	**F(30,60) = 11.92**	
	p**=**0.6018	p=0.0916	p=0.9575	**p<0.0001**	
B – Fig. 3B	Bias metric	Group	Group x metric	Participant	
CVC	**F(2,60) = 6.69**	F(2,60) = 2.33	F(4,60) = 0.45	**F(30,60) = 4.03**	
	**p=0.0024**	p=0.1150	p=0.7721	**p<0.0001**	
Tones	**F(2,60) = 19.1**	F(2,60) = 0.06	F(4,60) = 0.99	**F(30,60) = 1.72**	
	**p<0.0001**	p=0.9445	p=0.4184	**p=0.0371**	
Oddball	**F(2,60) = 3.61**	F(2,60) = 0.18	F(4,60) = 0.62	**F(30,60) = 8.2**	
	**p=0.033**	p=0.8344	p=0.6516	**p<0.0001**	
C	Rule	Run	Group	Run x Complexity	Run x Group
CVC	**F(2,890) = 141.2**	**F(2,890) = 28.46**	**F(30,890) = 14.4**	F(4,890) = 0.87	F(4,890) = 0.65
Fig. 3C	**p<0.001**	**p<0.001**	**p=0.029**	p=0.626	p=0.479
Tones	**F(2,890) = 73.8**	F(2,890) = 2.91	**F(30,890) = 4.26**	F(4,890) = 0.73	**F(4,890) = 3.34**
Fig. 3D	**p<0.001**	P=0.055	**p=0.026**	p=0.572	**p=0.010**

The tone language discriminability measured by A′ showed significant main effects of rule complexity and group, but no group x rule interaction or consistent inter-individual differences (Fig. 3B, Table 3A). For this language, post-hoc comparison of marginal means indicated that the group difference is driven by the control participants performing significantly better than participants in the nfvPPA group (p=0.048). Participants with stroke performed at an intermediate level, and were not statistically different from either controls (p=0.31) or nfvPPA (p=0.57). Rather than showing lower performance with increasingly complex rule violations, all groups performed significantly more poorly at detecting violations of the configurational rules than both the linear (p=0.0009) and hierarchical (p=0.0001), which in turn did not differ (p=0.73). Possible reasons for this unexpected pattern are discussed below.

All groups performed well at discriminating the oddball stimuli. For all groups, mean and median discriminability was better than for the CVC or tone languages. Crucially, there was no effect of rule type for the oddball language (Table 3A). This is because violations were no longer based on detection of grammatical rules, but on the detection of novel CVC tokens in various positions within the sequence. This observation reassures us that the effect of rule complexity in the CVC language cannot be explained by the position of the violation within a sequence. There was no significant group difference in performance or group x rule interaction (Table 3A), but there was a strongly significant effect of participant, indicating that individuals within each group differed in their ability to detect the novel non-word tokens. Post-hoc comparison of marginal means was not performed, as there was no rationale from the ANOVA to proceed to this.

**Fig. S3:**
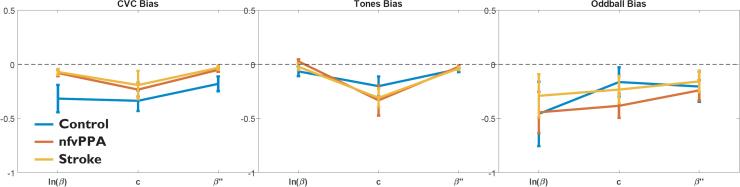
Bias metrics by group for each language type (values less than zero indicate a bias towards classifying sequences as correct).

From Fig. 3A it can be seen that for some sequences, particularly those which violate the more complex rules, participants in all groups performed at a level below chance (50% correct). This would not be expected if participants were simply guessing for these sequences, but would be expected if the grammatical violation was sufficiently subtle that participants made an active decision that the sequence is not inconsistent with the grammar. In other words, participants would display bias towards stating that a sequence was consistent with the artificial grammar unless they had evidence otherwise, but it was not known whether this tendency would differ between groups (Haendiges et al., 1996, Dickey et al., 2008). A bias towards yes (consistent) was demonstrated, but there was no group difference in bias or group x metric interaction (Table 3B, Supplementary Figure 3).

Participants, including patients, improved with practice. Results of the general linear model analysis including run number are illustrated in Fig. 3C, D, and Table 3C. For the CVC language, there were main effects of rule type, run number and group, but no interactions between run number and either rule complexity or group. This suggests, (a) that all groups were learning across repeated exposure-test cycles, (b) that the amount of learning over time was not different between the three rule types, and (c) that the group difference in overall discriminability described above was driven by differences in initial grammatical learning, not by a reduced ability to refine the internal grammatical model through feedback and repeated exposure. Tukey tests confirmed that participants improved on each set of exposure and testing (p<0.05 in all cases). Mean A′ across groups for run 1 was 0.55, for run 2 was 0.60, and for run 3 was 0.64 (chance performance is 0.5). Control performance was significantly superior to nfvPPA performance, which was in turn significantly superior to performance in the stroke group.

For the tone language, there were main effects of rule type and group, but only a trend towards a main effect of run number. There was an interaction between run number and group, but not run number and rule complexity. Tukey tests demonstrated that control participants performed significantly better than those with stroke, who in turn performed significantly better than those with nfvPPA. Performance on run 3 was significantly better than on run 1, while run 2 did not differ from either run 1 or 3.

Spearman rank-order correlograms are shown in Fig. 4A, and the resultant hierarchical cluster analysis is visualised in Fig. 4B. Rule acquisition patterns are highly correlated between groups within each task, but not between tasks within each group. This is confirmed by the cluster analysis. Performance on CVC and tone languages represent strongly distinct clusters, while oddball performance profiles are less distinct (as expected if sequence ordering is unimportant for novel token detection). The difference in profiles between CVC and tone languages across group was further assessed by a two-sample *t*-test with unequal variance based on the similarities shown in Fig. 4A. In sample 1 were the six within-language similarities (excluding the diagonal) and in sample 2 were the 9 between-language similarities. This confirmed a highly significant group difference in language similarity; t(12.5)=5.2, p=0.0002. Identical pair-wise tests between groups across language (blinded to overall ability by non-parametric Spearman rank-order correlation) confirmed that performance profiles did not differ between groups (control vs nfvPPA t(12.3)=−0.12, p=0.90; control vs stroke t(10.2)=−0.76, p=0.46; nfvPPA vs stroke t(12.2)=−0.01, p=0.99).

Stepwise regression analysis between overall measures of discriminability and the behavioural measures listed in the methods yielded no statistically significant predictors for CVC or oddball language performance. If performance on CVC linear rules (where performance was highest) is considered in isolation, overall aphasia severity (α=0.002) was the only significant predictor. The only significant predictor for tone language performance was diagnosis (α=0.035), confirming that patients with nfvPPA performed more poorly than those with stroke, independent of aphasia severity.

Lesion volume and site affected performance in the stroke group. Stepwise regression analysis between overall measures of discriminability for each language and the lesion metrics listed in methods yielded a model for the CVC language including only left putamen (α=0.06); in other words, the ability to detect sequencing violations decreased with more severe putaminal lesions. For the oddball language, total lesion volume (α=0.016) and involvement of the left ventral frontal operculum (α=0.076) were included in the model (overall p=0.023), but in opposite directions: participants with larger lesions were less able to detect oddball CVCs, but this deficit was ameliorated if their lesion had a more anterior distribution. The model yielded no statistically significant predictors for the tones language.

Grey matter volume affected performance in the nfvPPA group. The best model for nfvPPA performance on the CVC language included age (α=0.002) and total grey matter volume in the left frontal lobe regions of interest (α=0.016), but not in their right sided equivalents or total grey matter volume. These acted such that performance improved with higher left frontal grey matter volume, and also with age. While it might initially seem counter intuitive that older patients performed better, this is likely to reflect the natural loss of grey matter with age. The model is therefore improved by accounting for the fact that any given value of grey matter volume is relatively more atrophic in a younger individual. There were no statistically significant predictors with grey matter volume in the nfvPPA group for performance on the tone or oddball languages.

## 4 Discussion

This study successfully used a mixed-complexity artificial grammar learning task with speech sounds and tone stimuli to test aphasic patients with two different aetiologies. The principal observations were that: 1) both healthy individuals and patients with aphasia apply strongly contrasting strategies to assess structured sequences depending on whether the sequences consist of linguistic or non-linguistic auditory tokens; 2) patients with vascular aphasia and nfvPPA show similar patterns of auditory sequence processing impairment compared to controls; 3) aphasic patients are not disproportionately impaired on more complex auditory sequencing tasks, instead displaying a general impairment in processing structured auditory input; 4) patients with aphasia are capable of implicit learning of this kind through repeated exposure/test cycles. We discuss these results in turn in the following sections.

### 4.1 Rule acquisition differs when structurally identical sequences are comprised of linguistic or non-linguistic stimuli

In all groups, performance profiles on the CVC language followed the expected pattern of linear relationships being more discriminable than configurational or hierarchical structures. By contrast, participants’ judgments about the tone language did not seem be based on the abstraction of the intended grammatical rules (Fig. 3A, panel 2). This impression was confirmed by correlation and cluster analyses (Fig. 4). Hierarchical clustering based on non-parametric correlations of single subject performance profiles (Fig. 4B) was clearly able to recover the language learned, but not the group structure. This demonstrates that all groups acquired the same set of rules when making decisions about the CVC language; all that differed between groups was their overall performance. Further, a completely different set of rules were acquired for the tone language, but again this learning profile was almost identical across groups. Therefore, rule acquisition was not transferred between the two languages, and the separation of approach to linguistic and non-linguistic structured sequences was strongly maintained in agrammatic aphasia of either type. This is despite the two languages having an identical structure, and being presented and tested in the same order. This finding cannot be trivially explained by a lower level deficit such as the tone language simply being more difficult, more affected by a reduced fidelity of auditory processing or subject to a higher ‘lapse rate’, which would affect discriminability but could not produce the complete dissociation of response patterns shown here across all groups.

Despite extensive exposure, all groups performed poorly at classifying tone sequence number 1 as consistent with the artificial grammar, and indeed seemed to actively reject it, with control performance for this sequence well below chance. This sequence was comprised entirely of tone sweeps, embedded within a recursive structure. The participants also correctly classified sequence 12, which has similar properties, as inconsistent with the artificial grammar. In this case, good performance on this tone sequence does not reflect an ability to extract the hierarchical rule, but rather a consistent tendency to reject the embedded pattern of tone sweep sequences. Overall, therefore, it does not seem that the tone sequences were assessed for the specific violations of the grammatical rules inherent in the artificial language. Instead, they were judged on the overall ‘feel’ of the sequence in a manner very different to the CVC language but entirely consistent across groups.

We therefore infer that, while both patient groups are impaired in their ability to learn and discriminate sequencing rules for both CVC and tone languages, they maintain the same separation of processing of these languages seen in control participants. Taken together, these results imply that domain specific processes exist for linguistic and non-linguistic structured sequence learning, which are preserved even in the presence of acquired grammatical deficits. These processes might therefore engage different brain networks (Geranmayeh et al., 2014a, Geranmayeh et al., 2014b). It is possible that this separation is instantiated by an assessment of phonological ‘well-formedness’ in auditory temporal regions (Obrig et al., 2016).

### 4.2 Artificial grammar learning in aphasia is similar across aetiologies

A second key observation was that patients with nfvPPA and stroke showed similar patterns of performance for the CVC language. All groups were able to correctly classify the grammatical testing sequences as consistent with the exemplary sequences heard during exposure, and this ability fully generalised from the exposure set to the novel sequences not heard during exposure (Table 2; Fig. 3A). While there was a group difference, with performance in the stroke group being poorer on the CVC task, this effect disappeared when overall aphasia severity was accounted for. This novel result in agrammatic patients with primary progressive aphasia provides the evidence to suggest that existing findings on stroke patients should prove applicable to the progressive aphasias.

The lack of an effect of rule type for the oddball task confirms that the pattern of performance for the CVC language cannot be explained by stimulus-level differences such as the position of the violation within the sequence. The lack of a statistical group difference in the ability to detect oddball CVCs, a task performed at the end of the testing session, suggests that the patients’ impairments in this study are not solely due to generic difficulties with performing psychophysical sequence processing tasks, latent yes/no confusion, difficulties with basic auditory processing or differential effects of fatigue between groups.

The only consistent group difference not accounted for by severity was that patients with nfvPPA performed more poorly than those with stroke on the tone based language. There are a number of possible reasons for this. It might be a consequence of the tone language being more affected by the basic auditory sequence processing deficits previously demonstrated in nfvPPA (Goll et al., 2010, Grube et al., 2016). Alternatively, it might reflect involvement of the right IFC, which was spared in the stroke group (Fig. 1B), and is posited to have a role in prosodic and tonality based judgments. Nonetheless, patients with nfvPPA maintained the same pattern of learning as the other groups (Fig. 4), demonstrating that this deficit is a specific difficulty with processing tonal input rather than a breakdown of the separation of phonological vs tonal structured sequence processing.

### 4.3 Agrammatic aphasic patients are similarly impaired for both complex and simple sequencing operations

As expected, patients did not perform as well as controls, but the magnitude of this performance deficit did not differ by rule complexity, counter to our initial hypothesis. The results demonstrate that aphasic patients showed a global deficit in sequence processing, rather than a selective impairment on complex sequences. Clinically, patients in both groups make errors in the parsing of more complex syntax, and tend to stick to active, subject-relative structures in their expressive language (Grossman and Moore, 2005). We suggest that this does not reflect a specific deficit in the processing of more complex linguistic structures, but rather that these constructions are simply more difficult and therefore more vulnerable to a global deficit. As well as explaining a clinical symptom, this conclusion is consistent with the functional imaging finding that, in nfvPPA, left inferior frontal cortex activity lacks the normal relationship with syntactic complexity (Wilson et al., 2010); it suggests that the efficiency of IFC is so degraded that even the least complex grammatical structures require maximal neural recruitment. This is analogous to the finding that older adults are no longer able to selectively modulate anterior cingulate cortex in response to increasingly difficult listening environments as they have already fully engaged this region in easy listening conditions (Erb and Obleser, 2013).

It is possible that patients attempt to compensate for this deficit by engaging a wider syntactic processing network involving temporo-parietal regions (Schofield et al., 2012, Blank et al., 2016), but that this is insufficient to compensate for lost language function (Wilson et al., 2016). In stroke, where the IFC is lost entirely, the presence of residual ability could be due to complete reliance on this wider network (Thompson et al., 2010), or the involvement of contralateral IFC. Future functional imaging studies of implicit grammar learning will inform this debate. In any case, our findings of a general impact on sequence processing suggest that the grammatical deficits observed in aphasia, and the recovery from these, might reflect higher level, domain general processes (Caramazza and Zurif, 1976, Dominey et al., 2003, Patel et al., 2008, Brownsett et al., 2014, Geranmayeh et al., 2014a, Geranmayeh et al., 2014b, Geranmayeh et al., 2017).

The lack of group by rule complexity interactions is unlikely to be explained by limitations in sample size, as in no case was there even a trend in this direction (all interactions with complexity in Table 3 have p-values > 0.5). Therefore, these results appear to represent a genuine null effect, rather than sub-threshold effects that might become significant with a greater sample size. Moreover, the lack of interaction is also consistent in the tone and CVC experiments, although the response patterns between the two are vastly different. Nor can the results be trivially explained by floor effects in processing the more complex relationships for the following reasons: 1) the nfvPPA group show strikingly parallel behaviour in relation to the control group, consistent with a proportional impairment even on the linear sequencing operation (Fig. 3B, panel 1); 2) the stroke patients may well have reached a floor in performance on the complex sequences in the nonsense word task, but even excluding this group did not cause the group by complexity interaction to approach significance; 3) all groups improved over the three testing runs, and performance improvement was parallel in relation to sequencing complexity (i.e. there was a main effect of run but no run-by-complexity interaction, Figs. 3C); and 4) the tone language showed a very different pattern of results to that for the nonsense words yet, again, there was no evidence of a group-by-complexity interaction.

### 4.4 . **Patients with aphasia show improved performance over repeated cycles**

All three groups demonstrated the same amount of learning across repeated exposure/test cycles of the CVC language; the only difference was in their initial levels of performance (Fig. 3C; Table 3C). This suggests that patients with aphasia were able to update their internal model of the artificial grammar based on feedback and implicit comparison with short periods of exposure. It also suggests that patients did not suffer greater effects of fatigue than controls. By contrast, learning did not occur to the same degree for the tone language (Fig. 3D; Table 3C). It is therefore clear that learning was not transferrable between the CVC and tone languages, despite them sharing the same underlying artificial grammar structure.

Together, these findings provide a theoretical basis upon which an exposure-based speech therapy for grammar could be built with the aim of improving subjective difficulty with speech comprehension (Supplementary Figure 1). The results imply that there is potential for improvement from an intensive paradigm based on repeated exposure-test cycles. This could in principle be made home-deliverable and patient-led, an approach that has demonstrable efficacy for improving speech production in similar patient groups (Varley et al., 2016); the tasks employed in this study were automated and computer based. The finding that learning did not generalise across modalities implies that such a therapy would need to use linguistic material, as it would be unlikely to be so well learnt with non-linguistic material or to transfer across domains. In contrast, the finding that patients were able to generalise perfectly from sequences heard during exposure (Fig. 3A, sequences 1–3), to those that were novel during the test phase (Fig. 3A, sequences 4–6), to the extent that performance did not differ, suggests that such a therapy might not need to be comprehensive with regards to specific sentence structures. Instead, it is envisaged that a graded programme could be designed, such that training focusses initially on those structures that are having most frequent impact on speech comprehension. Clearly our study does not provide evidence that such a therapy would be more efficacious than existing methods for addressing asyntactic deficits after stroke, nor do we have any evidence of how well our strategies would work within an already-learned but now-impaired natural language. Indeed, a recent small study of nine patients who had chronic agrammatic aphasia secondary to stroke suggests that implicit learning alone (on a visuo-motor serial reaction time task) does not necessarily translate into improved real-world performance (Schuchard et al., 2017). Larger scale trials and assessment with other tasks are clearly required in more acute disease cohorts. As our method relies on exposure-based implicit learning rather than explicit instruction, future therapeutic trials could follow recent trends for patient-led practice in the home environment, increasingly with support from internet-based resources (Rogalski et al., 2016). This has the potential for providing the benefits of an intensive approach (Bhogal et al., 2003, Brady et al., 2016, Breitenstein et al., 2017) without the resource constraints that limit the frequency and therefore efficacy of traditional irregular, face-to-face instruction (Sarno et al., 1970, Lincoln et al., 1984).

### 4.5 Brain behaviour relationships

Our use of stepwise regression, to assess associations between brain structure and function, should be seen as exploratory, since the study was powered only to detect strong effect sizes. Nonetheless, significant associations between brain structure and behavioural performance within the patient groups were observed. The nfvPPA group demonstrated significant grey and white matter loss in frontal regions bilaterally. In keeping with previous studies of expressive grammar (Rogalski et al., 2011), the discriminability of grammatical structure in the CVC language correlated with age-corrected loss of volume only in left frontal regions (but not similar right-sided regions or total corrected grey matter volume). No such association was found for the tone language or the oddball task.

In the stroke group there was catastrophic loss of fronto-temporal regions in the dominant hemisphere, but complete contralateral preservation. For the CVC language, the model included only the putamen. The putamen is known from the functional imaging literature to be important in implicit sequence detection and learning in healthy individuals (Grafton et al., 1995, Rauch et al., 1995, Rauch et al., 1997, Grahn and Rowe, 2009). No such relationships were significant for the tone task. Performance on the oddball sound detection task was predicted by overall lesion volume and a weaker, opposite, effect of the extent of involvement of the most frontal region analysed. This suggests that more anterior lesion locations were less deleterious to CVC oddball detection than those located closer to auditory cortex in temporal lobe.

Similar performance in a relatively bilateral disease (nfvPPA) and one so clearly unilateral (stroke) immediately poses the question of which components of a broader language network are recruited to underpin the demonstrated learning over repeated exposure-test cycles (Crinion and Price, 2005). Thus, there is clear scope for a functional imaging study to be conducted in these patient groups, the results of which could complement the development of grammar-based speech therapy.

## 5 Conclusion

In this paper we reconcile a controversy in the literature regarding the effects of structural complexity on receptive grammar in the frontal aphasias. We demonstrate that, while the patients found complex, non-adjacent structuring relationships more difficult to acquire, this did not represent a disproportionate impairment; aphasia resulted in a similar performance penalty for adjacent relationships. We also provide insights into the language-specificity of artificial grammar learning by demonstrating that humans learn otherwise identical linguistic and non-linguistic structured sequences entirely separately, even if the neural architecture underlying this learning is disrupted. Our direct comparison of two patient groups suggests that previous findings regarding implicit sequence learning in stroke aphasia are likely to prove transferrable to nfvPPA. Finally, the ability of both patient groups to learn an artificial grammar as demonstrated here provides a rationale and approach for future trials of implicit, exposure-based approaches to rehabilitation of agrammatism in non-fluent aphasia.

## References

[bib1] Bahlmann J., Schubotz R.I., Friederici A.D. (2008). Hierarchical artificial grammar processing engages Broca's area. Neuroimage.

[bib2] Bastiaanse R., Edwards S., Mass E., Rispens J. (2003). Assessing comprehension and production of verbs and sentences: the Verb and Sentence Test (VAST). Aphasiology.

[bib3] Berndt R.S. (2000). Sentence comprehension in Broca's aphasia: a critique of the evidence. Behav. Brain Sci..

[bib4] Berndt R.S., Caramazza A., Zurif E. (1983). Language Functions: Syntax.

[bib5] Berndt R.S., Mitchum C.C., Haendiges A.N. (1996). Comprehension of reversible sentences in “agrammatism”: a meta-analysis. Cognition.

[bib6] Bhogal S.K., Teasell R.W., Foley N.C., Speechley M.R. (2003). Rehabilitation of aphasia: more is better. Top. Stroke Rehabil..

[bib7] Blank I., Balewski Z., Mahowald K., Fedorenko E. (2016). Syntactic processing is distributed across the language system. Neuroimage.

[bib8] Brady M.C., Kelly H., Godwin J., Enderby P., Campbell P. (2016). Speech and language therapy for aphasia following stroke. Cochrane Libr..

[bib9] Breitenstein C., Grewe T., Flöel A., Ziegler W., Springer L., Martus P. (2017). Intensive speech and language therapy in patients with chronic aphasia after stroke: a randomised, open-label, blinded-endpoint, controlled trial in a health-care setting. Lancet.

[bib10] Brownsett S.L., Warren J.E., Geranmayeh F., Woodhead Z., Leech R., Wise R.J. (2014). Cognitive control and its impact on recovery from aphasic stroke. Brain.

[bib11] Caplan D., Baker C., Dehaut F. (1985). Syntactic determinants of sentence comprehension in aphasia. Cognition.

[bib12] Caramazza A., Zurif E.B. (1976). Dissociation of algorithmic and heuristic processes in language comprehension: evidence from aphasia. Brain Lang..

[bib13] Christiansen M.H., Kelly M.L., Shillcock R.C., Greenfield K. (2010). Impaired artificial grammar learning in agrammatism. Cognition.

[bib14] Conway C.M., Bauernschmidt A., Huang S.S., Pisoni D.B. (2010). Implicit statistical learning in language processing: word predictability is the key. Cognition.

[bib15] Conway C.M., Pisoni D.B. (2008). Neurocognitive basis of implicit learning of sequential structure and its relation to language processing. Ann. N. Y Acad. Sci..

[bib16] Cope T.E., Patterson K., Sohoglu E., Dawson K., Grube M., Davis M.H. (2014). P67. Predictive mechanisms and speech perception in progressive non-fluent aphasia. Am. J. Neurodegener. Dis..

[bib17] Crinion J., Price C.J. (2005). Right anterior superior temporal activation predicts auditory sentence comprehension following aphasic stroke. Brain.

[bib18] Dickey M.W., Milman L.H., Thompson C.K. (2008). Judgment of functional morphology in agrammatic aphasia. J. Neurolinguist..

[bib19] Dominey P.F., Hoen M., Blanc J.-M., Lelekov-Boissard T. (2003). Neurological basis of language and sequential cognition: evidence from simulation, aphasia, and ERP studies. Brain Lang..

[bib20] Erb J., Obleser J. (2013). Upregulation of cognitive control networks in older adults' speech comprehension. Front Syst. Neurosci..

[bib21] Evans J.L., Saffran J.R., Robe-Torres K. (2009). Statistical learning in children with specific language impairment. J. Speech Lang. Hear Res..

[bib22] Fedorenko E., Duncan J., Kanwisher N. (2012). Language-selective and domain-general regions lie side by side within Broca's area. Curr. Biol..

[bib23] Folia V., Forkstam C., Ingvar M., Hagoort P., Petersson K.M. (2011). Implicit artificial syntax processing: genes, preference, and bounded recursion. Biolinguistics.

[bib24] Forkstam C., Hagoort P., Fernandez G., Ingvar M., Petersson K.M. (2006). Neural correlates of artificial syntactic structure classification. Neuroimage.

[bib25] Friederici A.D. (2011). The brain basis of language processing: from structure to function. Physiol. Rev..

[bib26] Friederici A.D., Bahlmann J., Heim S., Schubotz R.I., Anwander A. (2006). The brain differentiates human and non-human grammars: functional localization and structural connectivity. Proc. Natl. Acad. Sci. USA.

[bib27] Friederici A.D., Kotz S.A. (2003). The brain basis of syntactic processes: functional imaging and lesion studies. Neuroimage.

[bib28] Friederici A.D., Opitz B., von Cramon D.Y. (2000). Segregating semantic and syntactic aspects of processing in the human brain: an fMRI investigation of different word types. Cereb. Cortex.

[bib29] Frost R., Armstrong B.C., Siegelman N., Christiansen M.H. (2015). Domain generality versus modality specificity: the paradox of statistical learning. Trends Cogn. Sci..

[bib30] Geranmayeh F., Brownsett S.L., Wise R.J. (2014). Task-induced brain activity in aphasic stroke patients: what is driving recovery?. Brain.

[bib31] Geranmayeh F., Chau T., Wise R., Leech R., Hampshire A. (2017). Domain general sub-regions of the medial prefrontal cortex contribute to recovery of language after stroke. Brain.

[bib32] Geranmayeh F., Wise R.J., Mehta A., Leech R. (2014). Overlapping networks engaged during spoken language production and its cognitive control. J. Neurosci..

[bib33] Goll J.C., Crutch S.J., Loo J.H.Y., Rohrer J.D., Frost C., Bamiou D.E. (2010). Non-verbal sound processing in the primary progressive aphasias. Brain.

[bib34] Gómez R.L., Gerken L. (2000). Infant artificial language learning and language acquisition. Trends Cogn. Sci..

[bib35] Goodglass H., Barresi B., Kaplan E. (1983). The Boston Diagnostic Aphasia Examination.

[bib36] Goodman E., Bates J.C. (1997). On the inseparability of grammar and the lexicon: evidence from acquisition, aphasia and real-time processing. Lang. Cogn. Process..

[bib37] Gorno-Tempini M.L., Dronkers N.F., Rankin K.P., Ogar J.M., Phengrasamy L., Rosen H.J. (2004). Cognition and anatomy in three variants of primary progressive aphasia. Ann. Neurol..

[bib38] Gorno-Tempini M.L., Hillis A.E., Weintraub S., Kertesz A., Mendez M., Cappa S.F. (2011). Classification of primary progressive aphasia and its variants. Neurology.

[bib39] Grafton S.T., Hazeltine E., Ivry R. (1995). Functional mapping of sequence learning in normal humans. J. Cogn. Neurosci..

[bib40] Grahn J.A., Rowe J.B. (2009). Feeling the beat: premotor and striatal interactions in musicians and nonmusicians during beat perception. J. Neurosci..

[bib41] Grodzinsky Y. (2000). The neurology of syntax: language use without Broca's area. Behav. Brain Sci..

[bib42] Grossman M., Moore P. (2005). A longitudinal study of sentence comprehension difficulty in primary progressive aphasia. J. Neurol. Neurosurg. Psychiatry.

[bib43] Grube M., Bruffaerts R., Schaeverbeke J., Neyens V., De Weer A.S., Seghers A. (2016). Core auditory processing deficits in primary progressive aphasia. Brain.

[bib44] Grube M., Kumar S., Cooper F.E., Turton S., Griffiths T.D. (2012). Auditory sequence analysis and phonological skill. Proc. Biol. Sci..

[bib45] Haendiges A.N., Berndt R.S., Mitchum C.C. (1996). Assessing the elements contributing to a "mapping" deficit: a targeted treatment study. Brain Lang..

[bib46] Hickok G., Poeppel D. (2007). The cortical organization of speech processing. Nat. Rev. Neurosci..

[bib47] Jenkinson M., Smith S. (2001). A global optimisation method for robust affine registration of brain images. Med Image Anal..

[bib48] Josephs K.A., Duffy J.R., Strand E.A., Whitwell J.L., Layton K.F., Parisi J.E. (2006). Clinicopathological and imaging correlates of progressive aphasia and apraxia of speech. Brain.

[bib49] Kaplan E. (1983). The Assessment of Aphasia and Related Disorders.

[bib50] Kertesz A., McCabe P. (1977). Recovery patterns and prognosis in aphasia. Brain.

[bib51] Kertesz A., McMonagle P., Blair M., Davidson W., Munoz D.G. (2005). The evolution and pathology of frontotemporal dementia. Brain.

[bib52] Knibb J.A., Kipps C.M., Hodges J.R. (2006). Frontotemporal dementia. Curr. Opin. Neurol..

[bib53] Knibb J.A., Xuereb J.H., Patterson K., Hodges J.R. (2006). Clinical and pathological characterization of progressive aphasia. Ann. Neurol..

[bib54] Lincoln N.B., McGuirk E., Mulley G.P., Lendrem W., Jones A.C., Mitchell J.R. (1984). Effectiveness of speech therapy for aphasic stroke patients. A randomised controlled trial. Lancet.

[bib55] Macmillan N.A., Kaplan H.L. (1985). Detection theory analysis of group data: estimating sensitivity from average hit and false-alarm rates. Psychol. Bull..

[bib56] Makuuchi M., Bahlmann J., Anwander A., Friederici A.D. (2009). Segregating the core computational faculty of human language from working memory. Proc. Natl. Acad. Sci. USA.

[bib57] Mandelli M.L., Vilaplana E., Brown J.A., Hubbard H.I., Binney R.J., Attygalle S. (2016). Healthy brain connectivity predicts atrophy progression in non-fluent variant of primary progressive aphasia. Brain.

[bib58] Mesulam M.M., Weintraub S., Rogalski E.J., Wieneke C., Geula C., Bigio E.H. (2014). Asymmetry and heterogeneity of Alzheimer's and frontotemporal pathology in primary progressive aphasia. Brain.

[bib59] Ni W., Constable R.T., Mencl W.E., Pugh K.R., Fulbright R.K., Shaywitz S.E. (2000). An event-related neuroimaging study distinguishing form and content in sentence processing. Cogn. Neurosci. J..

[bib60] Obrig H., Mentzel J., Rossi S. (2016). Universal and language-specific sublexical cues in speech perception: a novel electroencephalography-lesion approach. Brain.

[bib61] Opitz B., Kotz S.A. (2012). Ventral premotor cortex lesions disrupt learning of sequential grammatical structures. Cortex.

[bib62] Patel A.D., Iversen J.R., Wassenaar M., Hagoort P. (2008). Musical syntactic processing in agrammatic Broca's aphasia. Aphasiology.

[bib63] Patterson K., Graham N., Ralph M.A.L., Hodges J. (2006). Progressive non-fluent aphasia is not a progressive form of non-fluent (post-stroke) aphasia. Aphasiology.

[bib64] Petersson K.-M., Folia V., Hagoort P. (2012). What artificial grammar learning reveals about the neurobiology of syntax. Brain Lang..

[bib65] Petersson K.M., Folia V., Hagoort P. (2012). What artificial grammar learning reveals about the neurobiology of syntax. Brain Lang..

[bib66] Petersson K.M., Forkstam C., Ingvar M. (2004). Artificial syntactic violations activate Broca's region. Cogn. Sci..

[bib67] Price C.J., Seghier M.L., Leff A.P. (2010). Predicting language outcome and recovery after stroke: the PLORAS system. Nat. Rev. Neurol..

[bib68] Rauch S.L., Savage C.R., Brown H.D., Curran T., Alpert N.M., Kendrick A. (1995). A PET investigation of implicit and explicit sequence learning. Hum. Brain Mapp..

[bib69] Rauch S.L., Whalen P.J., Savage C.R., Curran T., Kendrick A., Brown H.D. (1997). Striatal recruitment during an implicit sequence learning task as measured by functional magnetic resonance imaging. Hum. Brain Mapp..

[bib70] Raven J.C., 1960. Guide to the standard progressive matrices: sets A, B, C, D and E: *HK L* ewis.

[bib71] Reber A.S. (1967). Implicit learning of artificial grammars. J. Verbal Learn. Verbal Behav..

[bib72] Rogalski E., Cobia D., Harrison T.M., Wieneke C., Thompson C.K., Weintraub S. (2011). Anatomy of language impairments in primary progressive aphasia. J. Neurosci..

[bib73] Rogalski E.J., Saxon M., McKenna H., Wieneke C., Rademaker A., Corden M.E. (2016). Communication bridge: a pilot feasibility study of Internet-based speech–language therapy for individuals with progressive aphasia. Alzheimer'S. Dement.: Transl. Res. Clin. Interv..

[bib74] Rohrer J.D., Rossor M.N., Warren J.D. (2012). Alzheimer's pathology in primary progressive aphasia. Neurobiol. Aging.

[bib75] Romberg A.R., Saffran J.R. (2013). All together now: concurrent learning of multiple structures in an artificial language. Cogn. Sci..

[bib76] Sajjadi S.A., Patterson K., Arnold R.J., Watson P.C., Nestor P.J. (2012). Primary progressive aphasia: a tale of two syndromes and the rest. Neurology.

[bib77] Sajjadi S.A., Patterson K., Nestor P.J. (2014). Logopenic, mixed, or Alzheimer-related aphasia?. Neurology.

[bib78] Sarno M.T., Silverman M., Sands E. (1970). Speech therapy and language recovery in severe aphasia. J. Speech Hear Res..

[bib79] Schofield T.M., Penny W.D., Stephan K.E., Crinion J.T., Thompson A.J., Price C.J. (2012). Changes in auditory feedback connections determine the severity of speech processing deficits after stroke. J. Neurosci..

[bib80] Schuchard J., Nerantzini M., Thompson C.K. (2017). Implicit learning and implicit treatment outcomes in individuals with aphasia. Aphasiology.

[bib81] Schuchard J., Thompson C.K. (2017). Sequential learning in individuals with agrammatic aphasia: evidence from artificial grammar learning. J. Cogn. Psychol..

[bib82] Seghier M.L., Patel E., Prejawa S., Ramsden S., Selmer A., Lim L. (2016). The PLORAS database: a data repository for predicting language outcome and recovery after stroke. Neuroimage.

[bib83] Stanislaw H., Todorov N. (1999). Calculation of signal detection theory measures. Behav. Res Methods Instrum. Comput..

[bib84] The Mathworks Inc (2015). Matlab and Statistics and Machine Learning Toolbox. Release.

[bib85] Thompson C.K., den Ouden D.-B., Bonakdarpour B., Garibaldi K., Parrish T.B. (2010). Neural plasticity and treatment-induced recovery of sentence processing in agrammatism. Neuropsychologia.

[bib86] Thompson C.K., Meltzer-Asscher A., Cho S., Lee J., Wieneke C., Weintraub S. (2013). Syntactic and morphosyntactic processing in stroke-induced and primary progressive aphasia. Behav. Neurol..

[bib87] Varley R., Cowell P.E., Dyson L., Inglis L., Roper A., Whiteside S.P. (2016). Self-administered computer therapy for apraxia of speech. Stroke.

[bib88] Wilson B., Smith K., Petkov C.I. (2015). Mixed-complexity artificial grammar learning in humans and macaque monkeys: evaluating learning strategies. Eur. J. Neurosci..

[bib89] Wilson S.M., DeMarco A.T., Henry M.L., Gesierich B., Babiak M., Miller B.L. (2016). Variable disruption of a syntactic processing network in primary progressive aphasia. Brain.

[bib90] Wilson S.M., Dronkers N.F., Ogar J.M., Jang J., Growdon M.E., Agosta F. (2010). Neural correlates of syntactic processing in the nonfluent variant of primary progressive aphasia. J. Neurosci..

[bib91] Wilson S.M., Galantucci S., Tartaglia M.C., Rising K., Patterson D.K., Henry M.L. (2011). Syntactic processing depends on dorsal language tracts. Neuron.

[bib92] Wong D.L., Baker C.M. (1988). Pain in children: comparison of assessment scales. Okla. Nurse.

[bib93] Zimmerer V.C., Cowell P.E., Varley R.A. (2014). Artificial grammar learning in individuals with severe aphasia. Neuropsychologia.

[bib94] Zimmerer V.C., Dąbrowska E., Romanowski C.A., Blank C., Varley R.A. (2014). Preservation of passive constructions in a patient with primary progressive aphasia. Cortex.

[bib95] Zimmerer V.C., Varley R.A. (2015). A case of “order insensitivity”? Natural and artificial language processing in a man with primary progressive aphasia. Cortex.

