# Supplementary material for: Artificial grammar learning in vascular and progressive non-fluent aphasias
Source: Neuropsychologia. 2017 Sep;104:201–13. doi: 10.1016/j.neuropsychologia.2017.08.022 (PMC5637161; doi:10.1016/j.neuropsychologia.2017.08.022)
Supplement: Supplementary file 1 — Supplementary Material [file mmc4.docx]

Supplementary table 1: Single subject lesion percentages by region for the stroke group.

| Subject Number | Broca WM | Broca GM | FOP WM | FOP GM | Frontal Inferior Trigone | Frontal Inferior Operculum | Rolandic Operculum | Putamen | Caudate |
| --- | --- | --- | --- | --- | --- | --- | --- | --- | --- |
| 1 | 99 | 98 | 99 | 97 | 98 | 98 | 92 | 74 | 28 |
| 2 | 32 | 33 | 30 | 19 | 33 | 22 | 46 | 77 | 17 |
| 3 | 0 | 0 | 8 | 45 | 0 | 34 | 83 | 3 | 0 |
| 4 | 77 | 86 | 77 | 89 | 84 | 86 | 89 | 70 | 42 |
| 5 | 77 | 54 | 88 | 84 | 60 | 85 | 89 | 10 | 47 |
| 6 | 100 | 96 | 94 | 61 | 97 | 70 | 71 | 12 | 59 |
| 7 | 89 | 91 | 85 | 89 | 91 | 88 | 100 | 16 | 0 |
| 8 | 0 | 0 | 21 | 3 | 0 | 8 | 26 | 51 | 36 |
| 9 | 54 | 32 | 97 | 73 | 38 | 80 | 99 | 76 | 10 |
| 10 | 16 | 1 | 35 | 8 | 5 | 16 | 0 | 59 | 30 |
| 11 | 79 | 59 | 92 | 62 | 65 | 70 | 48 | 48 | 46 |
| 12 | 32 | 5 | 30 | 16 | 20 | 13 | 21 | 0 | 0 |

Supplementary table 2: P-values for a repeated measures ANOVA of group against rule for the parametric discriminability measure d’, with participant number as a nested factor within group.

|  | Rule complexity | Group | Group x Rule | Participant |
| --- | --- | --- | --- | --- |
| CVC | **<0.0001** | **0.0311** | 0.6445 | **0.0006** |
| Tones | **0.0006** | **0.0027** | 0.6069 | 0.9994 |
| Oddball | 0.1383 | 0.0611 | 0.7245 | **<0.0001** |

Supplementary table 3. P-values for the general linear model assessing learning across runs for the parametric discriminability measure d’.

|  | Rule | Run | Group | Rule x Run | Run x Group |
| --- | --- | --- | --- | --- | --- |
| CVC | **<0.001** | **<0.001** | **0.020** | 1 | 0.054 |
| Tones | 0.070 | **<0.001** | **0.010** | 0.333 | **0.019** |
